# Long-Term Hepatitis B Virus (HBV) Response to Lamivudine-Containing Highly Active Antiretroviral Therapy in HIV-HBV Co-Infected Patients in Thailand

**DOI:** 10.1371/journal.pone.0042184

**Published:** 2012-07-31

**Authors:** Woottichai Khamduang, Catherine Gaudy-Graffin, Nicole Ngo-Giang-Huong, Gonzague Jourdain, Alain Moreau, Nuananong Luekamlung, Guttiga Halue, Yuwadee Buranawanitchakorn, Sura Kunkongkapan, Sudanee Buranabanjasatean, Marc Lallemant, Wasna Sirirungsi, Alain Goudeau, for the Program for HIV Prevention and Treatment (PHPT) study group

**Affiliations:** 1 Faculty of Associated Medical Sciences, Chiang Mai University, Chiang Mai, Thailand; 2 INSERM U966, CHRU Tours, Faculté de Médecine, Université François-Rabelais, Tours, France; 3 Institut de Recherche pour le Développement (IRD) UMI 174-Programs for HIV Prevention and Treatment (PHPT), Chiang Mai, Thailand; 4 Department of Immunology and Infectious Diseases, Harvard School of Public Health, Boston, Massachusetts, United States of America; 5 Lamphun Hospital, Lamphun, Thailand; 6 Phayao Hospital, Phayao, Thailand; 7 Chiang Kham Hospital, Phayao, Thailand; 8 Mae Sai Hospital, Chiangrai, Thailand; 9 Mae Chan Hospital, Chiangrai, Thailand; University of Modena & Reggio Emilia, Italy

## Abstract

**Background:**

Approximately 4 million of people are co-infected with HIV and Hepatitis B virus (HBV). In resource-limited settings, the majority of HIV-infected patients initiate first-line highly active antiretroviral therapy containing lamivudine (3TC-containing-HAART) and long-term virological response of HBV to lamivudine-containing HAART in co-infected patients is not well known.

**Methodology/Principal Finding:**

HIV-HBV co-infected patients enrolled in the PHPT cohort (ClinicalTrials.gov NCT00433030) and initiating a 3TC-containing-HAART regimen were included. HBV-DNA, HIV-RNA, CD4+ T-cell counts and alanine transaminase were measured at baseline, 3 months, 12 months and then every 6 months up to 5 years. Kaplan-Meier analysis was used to estimate the cumulative rates of patients who achieved and maintained HBV-DNA suppression. Of 30 co-infected patients, 19 were positive for HBe antigen (HBeAg). At initiation of 3TC-containing-HAART, median HBV DNA and HIV RNA levels were 7.35 log_10_ IU/mL and 4.47 log_10_ copies/mL, respectively. At 12 months, 67% of patients achieved HBV DNA suppression: 100% of HBeAg-negative patients and 47% of HBeAg-positive. Seventy-three percent of patients had HIV RNA below 50 copies/mL. The cumulative rates of maintained HBV-DNA suppression among the 23 patients who achieved HBV-DNA suppression were 91%, 87%, and 80% at 1, 2, and 4 years respectively. Of 17 patients who maintained HBV-DNA suppression while still on 3TC, 4 (24%) lost HBsAg and 7 of 8 (88%) HBeAg-positive patients lost HBeAg at their last visit (median duration, 59 months). HBV breakthrough was observed only in HBeAg-positive patients and 6 of 7 patients presenting HBV breakthrough had the rtM204I/V mutations associated with 3TC resistance along with rtL180M and/or rtV173L.

**Conclusions:**

All HBeAg-negative patients and 63% of HBeAg-positive HIV-HBV co-infected patients achieved long-term HBV DNA suppression while on 3TC-containing-HAART. This study provides information useful for the management of co-infected patients in resource-limited countries where the vast majority of co-infected patients are currently receiving 3TC.

## Introduction

In 2010, the World Health Organization (WHO) estimated that 34 million people were HIV infected worldwide [1]; of whom, approximately 4 million have chronic hepatitis B virus (HBV) infection (defined by more than 6 months of hepatitis B surface antigen or HBsAg in the blood) [2]. These HIV-HBV co-infected individuals are at risk of accelerated liver disease progression, aggressive hepatocellular carcinoma and 8-fold increased liver-related mortality rate [3], [4].

Current guidelines for the use of antiretroviral agents in HIV-1-infected patients [5], [6], [7], recommend to screen HBsAg in all HIV patients prior to initiating antiretroviral treatment (ART), and if found positive, initiate combination antiretroviral with a tenofovir disoproxil fumarate (TDF) plus lamivudine (3TC) or emtricitabine (FTC) backbone. However, since HBsAg testing has not been available or is too costly in many resource-limited settings, HBV infection status is unknown for the vast majority of HIV infected patients who have thus received a standard first line that includes 3TC as part of triple combination antiretroviral therapy.

Lamivudine is a cytidine analogue that inhibits the reverse transcriptase of both HIV and HBV [8], [9], [10]. However, its major drawback is the progressive emergence of resistance mutations at a rate of 15–20% per year in HBV-HIV-1 co-infected patients in developed countries [11], [12]. New generation nucleos(t)ide analogues with higher resistance barriers have been produced, in particular TDF for which no resistance mutation has been detected in chronically HBV infected patients after 3 years of therapy [13]. However, in resource-limited countries, most of these new drugs are not available or too expensive. Indeed, the 2008 Asian Pacific Association for the Study of the Liver (APASL), still recommends 3TC for treatment of HBV mono-infection in endemic areas [14].

In Thailand, since the national scale-up of ART in 2004 [15], over 95% of HIV-infected patients have received 3TC as part of highly active antiretroviral therapy (HAART) [16] and there are still numerous HIV-HBV co-infected patients on 3TC-containing HAART regimens for whom the long-term benefit of 3TC on HBV replication and the incidence of 3TC resistance mutations are not well known. We report the analysis of virological efficacy of 3TC on HBV replication and emergence of 3TC resistance HBV variants in HBV-HIV-coinfected patients receiving up to 5 years of 3TC-containing HAART regimens.

## Methods

### Patients

Patients were enrolled in the prospective multicenter Program for HIV Prevention and Treatment (PHPT) cohort (ClinicalTrials.gov Identifier: NCT00433030) of HIV-infected adults on antiretroviral therapy in Thailand and provided written informed consent at entry. This cohort study was approved by the Thai Ministry of Public Health and ethic committees at Chiangrai Prachanukroh, Prapokklao, Chonburi, Bhuddasothorn, Somdej Prapinklao, Nopparat Rajathanee, Bhumibol Adulyadej, Buddhachinaraj, Hat Yai, Samutsakorn, Nakhonpathom, Maharaj Nakornratchasrima, Sanpatong Hospitals. All investigators conducted the study according to the principles expressed in the Declaration of Helsinki. This sub-study was also approved by the ethic committee of the Faculty of Associated Medical Sciences, Chiang Mai University.

At entry, prior to starting HAART, all patients were screened for HBsAg and anti-HCV antibodies and had CD4+ T-cell count and plasma HIV RNA load performed. Following HAART initiation, CD4+ T-cell enumeration and plasma HIV RNA were measured every 6 months. Patients received a quarterly clinical biological follow-up and compliance was assessed at each visit by pill count and self-reporting.

Patients were included in this analysis if 1) HBsAg positive, 2) receiving HAART regimens which included 3TC (150 mg twice a day), 3) stored blood samples collected prior to 3TC initiation (baseline), and on HAART (3-month, 12-month, annual sample until up to 5 years) were available, and 4) HBV DNA was detectable at baseline.

### HBV Markers and HIV RNA Quantification

HBsAg and HBeAg were tested at baseline using DiaSorin ETI-MAK-4 and DiaSorin ETI-EBK PLUS (Salluggia, Italy), respectively. HBsAg was tested in patients on 3TC treatment using the MonoLisa® HBsAg ultra with a sensitivity of 50 pg/mL (Bio-Rad laboratories, France). HBV viral load was quantified using the Abbott real-time HBV DNA™ assay, Rungis, France (lower limit of detection 1.18 log_10_ IU/mL). HIV RNA was quantified using the COBAS Amplicor HIV-1 Monitor Test v.1.5. (Roche Molecular Systems, Branchburg, NJ) (lower limit of detection: 1.70 log_10_ copies/mL) and the Abbott real-time HIV RNA™ assay, Abbott, (lower limit of detection: 1.6 log_10_ copies/mL).

### HBV Virological Responses

HBV responses to 3TC were categorized according to the APASL recommendations [14]; HBV DNA suppression was defined as undetectable level of HBV DNA (the threshold used was 2.18 log_10_ IU/mL since some samples were diluted 1∶10 due to insufficient volume); virological breakthrough defined as an initial decline >2 log_10_ IU/mL followed by an increase of HBV DNA >1 log_10_ IU; and maintained viral suppression defined as HBV DNA level persistently <2.18 log_10_ IU/mL.

### HBV DNA Sequencing

All baseline samples as well as any sample with detectable HBV DNA on 3TC-containing-HAART were tested for HBV resistance mutation; HBV DNA sequencing was performed as previously described [17] with slight modification of PCR conditions. First round PCR conditions consisted of an initial 2 min denaturation step at 94°C, followed by 40 cycles of 1 min at 94°C for, 1 min at 48°C, and 3 min at 68°C. The second-round PCR included an initial denaturation step of 2 min at 94°C, followed by 30 cycles of 40 sec at 94°C, 1 min at 55°C, and 3 min at 68°C. The second-round PCR products were sequenced using the nested pol3M and pol4M primers and the BigDye Terminator Mix V. 1.1 (Applied Biosystems, Foster city, CA). Sequences were analyzed using the Bioedit software (http://www.mbio.ncsu.edu/bioedit) and HBV genotype was identified by phylogenetic analysis. HBV *pol* sequences were analyzed for polymorphisms and mutations known to be associated with 3TC resistance through comparison with wild-type reference sequences of similar genotype [18].

### Statistical Analyses

Baseline characteristics are reported as percentage with 95% confidence interval (95%CI) or medians with interquartile ranges (IQR) and were compared according to HBeAg status using Fisher’s exact test for categorical variables and Wilcoxon rank-sum test for continuous variables. Kaplan-Meier analysis was used to estimate the cumulative rates of achieving serum HBV DNA and, in those who achieved HBV DNA suppression, the duration of maintaining such suppression. Duration of HBV DNA suppression was counted from the date of first viral suppression until the date of first detectable HBV DNA or, if a patient was lost to follow-up or switched to a regimen without 3TC, the date of last undetectable HBV DNA load. Log-rank test was used to compare the cumulative rates of virological responses between HBeAg-positive and HBeAg-negative patients. For the analysis of association between the baseline CD4, HIV RNA, HBV DNA levels and virological responses to 3TC-containing HAART, continuous data were dichotomized according to the median baseline values and analysis was performed using Fisher’s exact or log-rank tests. Statistical significance was defined as p<0.05. Data were analyzed using STATA™ version 10.1 software (Statacorp, College Station, Texas, USA).

## Results

### Baseline Characteristics

**Figure 1:**
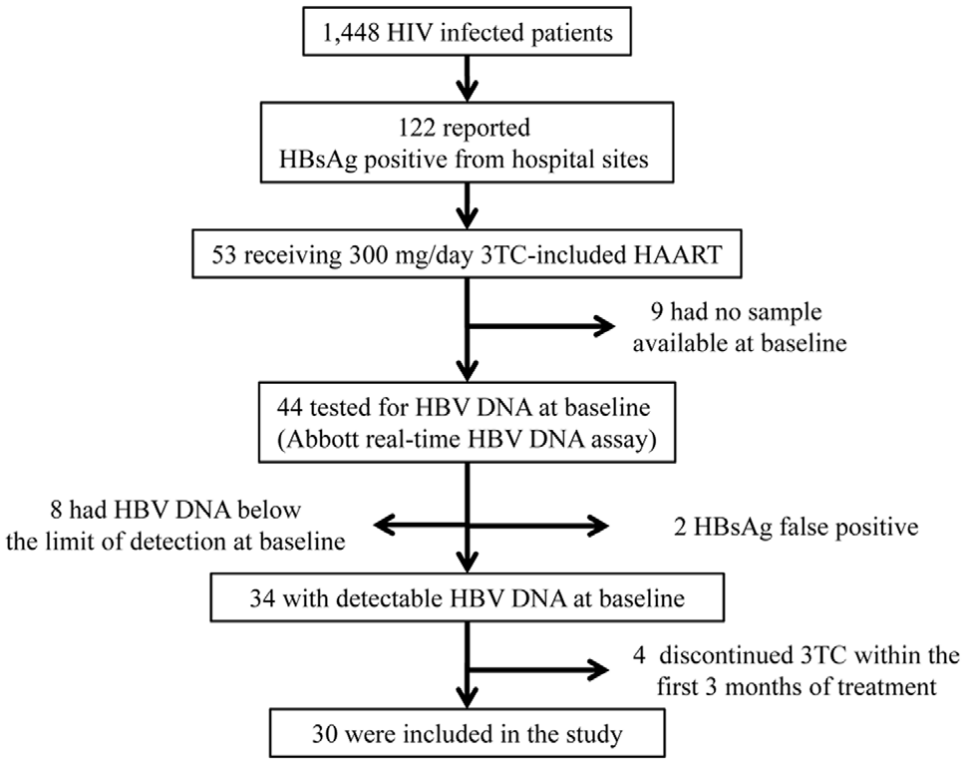
Disposition of patients included in this study.

Of 1,448 HIV infected adults on HAART, 122 (8.4%) tested HBsAg-positive. Thirty patients met all criteria for this analysis i.e. receiving 150 mg twice a day (bid) of 3TC as part of HAART, blood samples collected at baseline and on 3TC-based treatment and detectable HBV DNA at baseline (Figure 1). Nineteen patients (63%) were HBeAg-positive. Patients’ baseline characteristics were not different between HBeAg-positive and -negative patients (Table 1), except for the median HBV DNA level significantly higher, as expected, in HBeAg-positive patients and the alanine transaminase (ALT) level which tended to be higher in HBeAg-negative patients than in HBeAg-positive patients. Phylogenetic analysis indicated that 17% of patients were infected with HBV B genotype and 83% with C genotype (data not shown). None had HCV infection. Twelve patients (40%) had received previous antiretroviral treatment but none had been exposed to 3TC except one for a short period of 1 month. Over the course of 3TC-containing HAART, 29 of 30 patients had >95% adherence by pill count and 20 patients self-reported that they always missed ≤1 dose per week.

**Table 1:** Baseline demographic and clinical characteristics of the study population.

Baseline characteristics	Overall		HBeAg positive (N = 19)		HBeAg negative (N = 11)		p-value^a^
	n	Value	n	Value	n	Value	
Age (year) [median (IQR)]	30	31 (27–34)	19	29 (27–33)	11	33 (27–35)	0.59
Female [n (%)]	30	24 (80)	19	17 (89)	11	7 (64)	0.16
Treatment-experienced [n (%)]	30	12 (40)	19	7 (37)	11	5 (45)	0.71
CD4+ T-cell count (x10^6^/L) [median (IQR)]	30	100 (38–178)	19	110 (38–188)	11	48 (33–178)	0.78
HIV RNA (log_10_copies/mL) [median (IQR)]	30	4.47 (4.09–5.27)	19	4.46 (4.06–5.25)	11	5.25 (4.25–5.50)	0.29
Alanine transaminase (IU/L) [median (IQR)]	30	30 (20–39)	19	27 (17–36)	11	44 (21–121)	0.06
HBV DNA (log_10_IU/mL) [median (IQR)]	30	7.35 (5.55–8.07)	19	7.92 (7.34–8.31)	11	3.76 (3.28–6.67)	<0.001
HBV Genotype B : C [n (%)]	30	5∶25 (17∶83)	19	4∶15 (21∶79)	11	1∶10 (9∶91)	0.63

a Fisher’s exact test or Wilcoxon rank-sum test were used.

### Efficacy of 3TC on HBV Replication

After 3 month of HAART, median reduction of HBV DNA was 3.86 log_10_ (IQR, 2.56–4.67), and 47% (95%CI, 28–66) of patients achieved HBV DNA suppression. At 12 months, median HBV DNA reduction was 4.40 log_10_ (IQR, 2.89–5.65) IU/mL and 20 patients (67%; 95%CI, 47–83) of patients achieved HBV DNA suppression.

**Figure 2:**
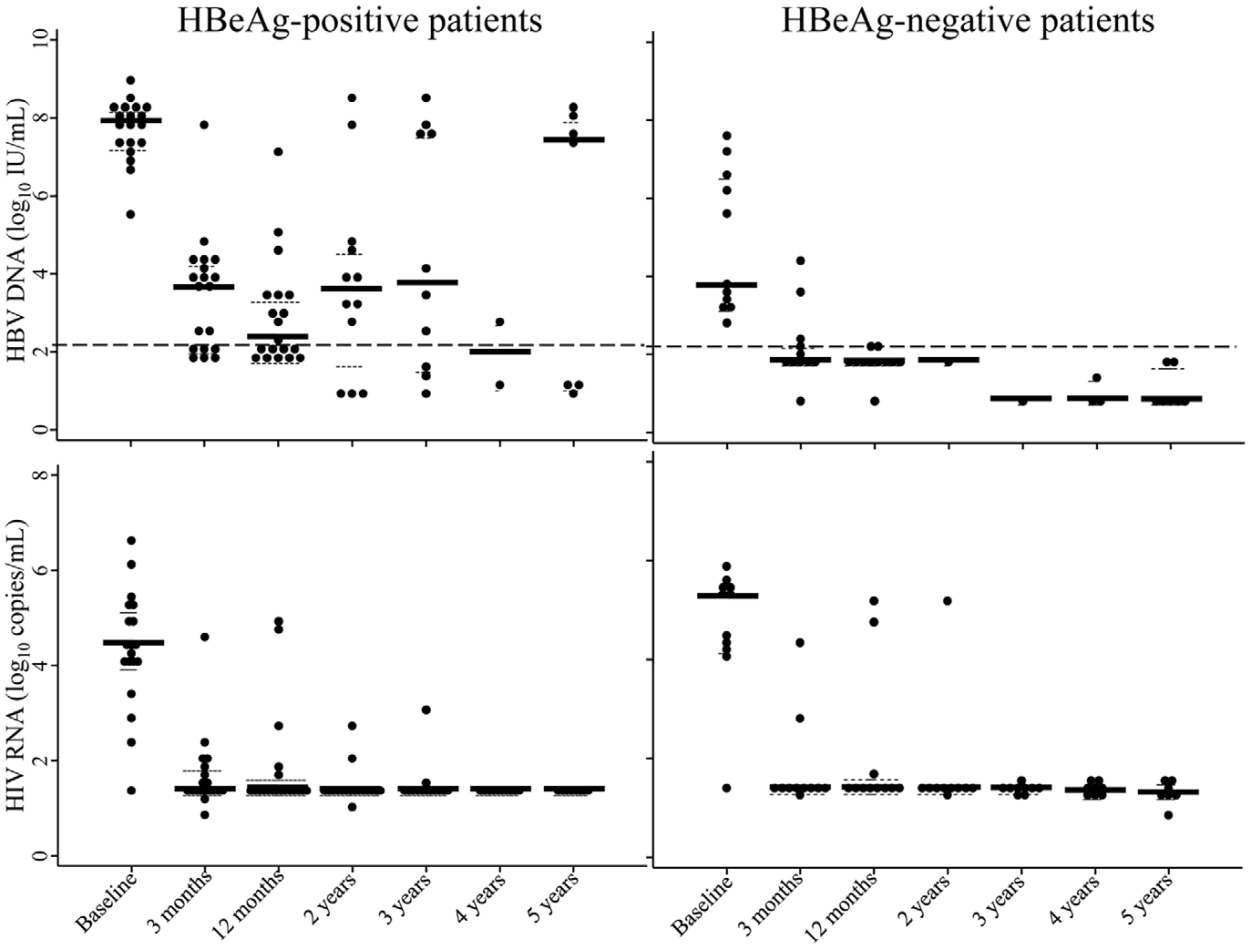
HBV DNA and HIV RNA load in HIV-HBV co-infected patients after initiation of 3TC-containing-HAART. Dotted line indicates the lower limit of detection for HBV DNA (2.18 log_10_ IU/mL).

The rate of HBV DNA suppression at 12 months was significantly higher among HBeAg-negative patients than among HBeAg-positive patients (100% and 47%, respectively; p = 0.004; Table 2 and Figure 2). Kaplan-Meier analysis showed that HBeAg-negative patients achieved HBV DNA suppression more rapidly than HBeAg-positive patients (p = 0.01). Six patients had partial HBV virological response and 4 experienced a HBV breakthrough during the first 12 months; of whom 2 had HBV DNA suppression at 3 months and 2 never fully suppressed HBV replication.

**Table 2:** HBV and HIV response to 3TC in HIV-1/HBV co-infected patients during 12 months of treatment.

	Overall (N = 30)		HBeAg positive (N = 19)		HBeAg negative (N = 11)		p-value^a^
	n	%[95%CI] or median [IQR]	n	%[95%CI] or median [IQR]	n	%[95%CI] or median [IQR]	
HBV DNA suppression^b^							
at 3 months	14	47 [28–66]	6	32 [13–57]	8	73 [39–94]	0.06
at 12 months	20	67 [47–83]	9	47 [24–71]	11	100 [72–100]	0.004
HIV load ≤50 cp/mL							
at 3 months	22	73 [54–88]	13	68 [43–87]	9	82 [48–98]	0.67
at 12 months	22	73 [54–88]	14	74 [49–91]	8	73 [39–94]	1.00
HIV RNA reduction							
at 3 months (log_10_ cp/mL)		2.92 [2.54–3.53]		2.93 [2.14–3.48]		2.91 [2.54–4.08]	0.53
at 12 months (log_10_ cp/mL)		2.92 [1.52–3.45]		2.93 [1.52–3.32]		2.91 [1.17–4.08]	0.78

a Fisher’s exact test or Wilcoxon rank-sum test were used.

b HBV DNA suppression was defined as serum HBV DNA level equal or below 150 or 2.18 log_10_ IU/mL.

Over 5 years of follow-up, 17 of 20 patients who achieved HBV DNA suppression at 12 months were able to control their HBV replication until their last medical visit (with a median of 59 months [IQR, 32–60]), 2 had a viral breakthrough and one had no long-term sample available.

Of 6 patients with partial HBV virological response at 12 months, one patient achieved slower HBV DNA suppression, at 38 months of treatment, 3 had detectable HBV DNA (i.e. 2.71, 2.84, and 4.76 log_10_ IU/mL), one had a viral breakthrough at 24 months of treatment, and one switched treatment regimen (Table S1).

To analyze the relation between baseline HIV RNA load and HBV virological response to 3TC-containing HAART, patient baseline HIV RNA levels were dichotomized according to the median baseline HIV RNA level (4.47 log_10_ copies/mL). The baseline HBV DNA levels did not differ between the two baseline HIV RNA groups (7.19 versus 7.42 log_10_ IU/mL, p = 0.31). At one year, there was no difference in HBV response (67% versus 67%) by baseline HIV RNA group. Furthermore, there was no difference in response during 5-years of 3TC-containing HAART (log-rank p-value  = 0.26).

**Figure 3:**
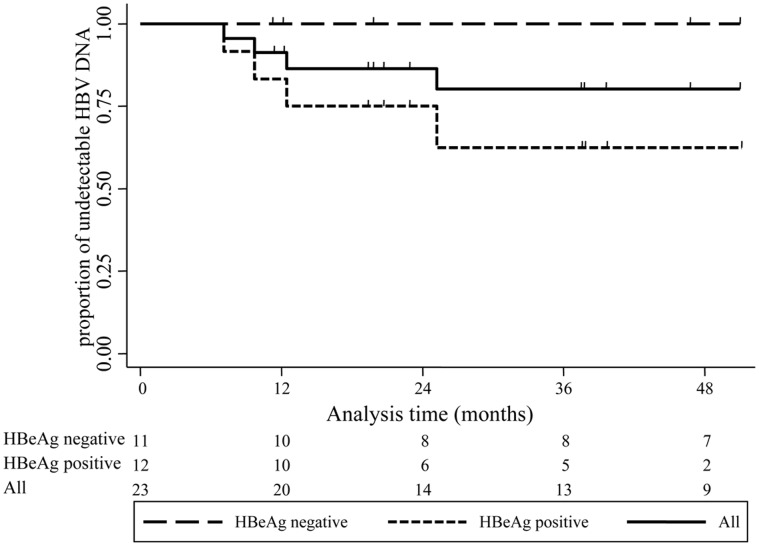
Kaplan-Meier curve of time to loss of HBV DNA suppression in 23 HIV-HBV co-infected patients who had achieved HBV DNA suppression during the course of 3TC-containing-HAART.

The duration of HBV DNA suppression was analyzed for the 23 patients who achieved HBV DNA suppression (i.e. 20 achieved HBV DNA suppression at 12 months, 2 had a viral breakthrough within 12 months and one who achieved viral suppression after 12 months of treatment). Eighteen (78%) maintained HBV DNA suppression up to their last visit, 4 (17%) had a HBV breakthrough, and one changed drug regimen after 15 months of 3TC treatment (Table S1). The cumulative rates of maintained HBV DNA suppression were estimated to be 91% (95%CI; 69–98), 87% (95%CI; 64–95), 80% (95%CI; 55–92) and 80% (95%CI; 55–92) at 1, 2, 3, and 4 years, respectively (Figure 3).

### HBV Serological Evaluations in Patients on 3TC-contained HAART

Of the 18 patients who maintained long-term HBV DNA suppression, 17 had a sample available for HBsAg testing. Four patients (24%) lost HBsAg at their last visit; one was HBeAg positive and three were HBeAg negative when initiating of 3TC-based HAART. Among the 8 HBeAg positive patients, 7 lost HBeAg at their last visit.

### 3TC Resistance-associated Mutations

There was no mutation associated with resistance to 3TC observed in all 30 patients before initiation of 3TC-containing-HAART. The 5-year cumulative rate of 3TC resistance was 20% (6/30); all of which was found in HBeAg positive patients. The mutations were found in 6 of 7 patients with viral breakthrough. Early viral breakthrough (within 1 year of therapy) occurred between 4 and 12 months in 4 patients. One patients had the HBV mutation ntG741A (G to A nucleotide at position 741), resulting in the known 3TC-associated resistance mutation rtM204I. This mutation led to the concomitant substitution of tryptophan to stop codon at position 196 (sW196stop) in the surface protein. The ntT843G mutation of unknown significance was also observed and resulted in the change from asparagine to lysine in the reverse transcriptase protein (rtN238K). In the 3 other patients, the viral breakthrough was not associated with the detection of HBV resistance mutations; however the M204V mutation was detected later during the follow-up in 2 patients, (i) at 42 months, along the compensatory resistance mutations rtV173L+L180M in one patient, and (ii) at 78 months with rtL180M mutation in one patient. No long-term HBV sequence was available for the third patient due to subsequent switch to non-3TC including regimen.

Late HBV breakthrough occurred in 3 patients and was associated with the emergence of 3TC-resistance mutations: rtV173L+L180M+M204I, rtL180M+M204V and rtL180M+M204I. Four patients never achieved undetectable HBV DNA but no resistance mutations were found before they switched to non-3TC regimen or recieved add-on TDF.

### Efficacy of HAART on HIV Replication, CD4 Cell Count and Alanine Transaminase Levels

**Figure 4:**
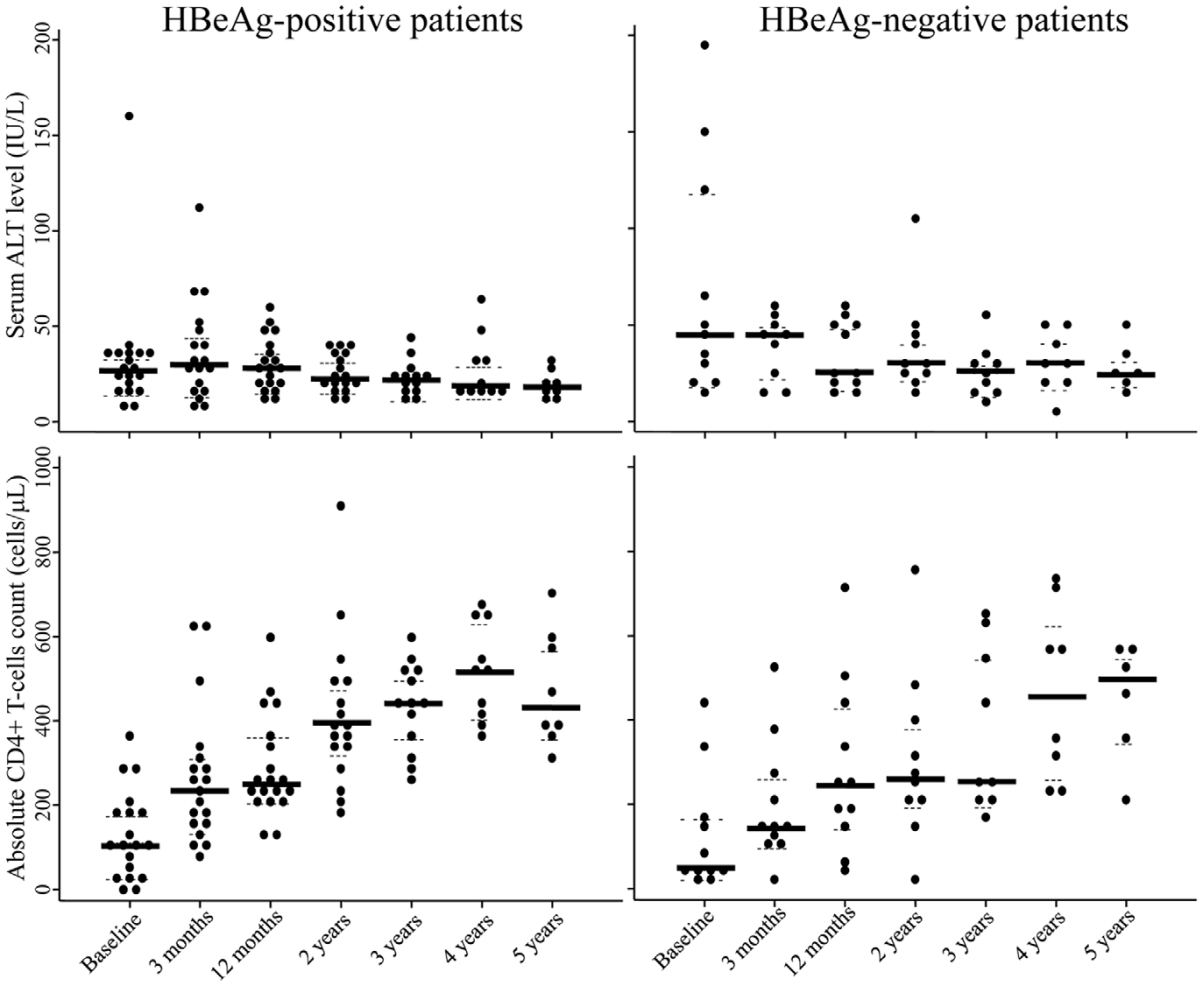
Serum ALT level and CD4+ T-cells count in HIV-HBV co-infected patients after initiation of 3TC-containing-HAART.

At 3-month, the median reduction of HIV RNA was 2.92 log_10_ (IQR, 2.54–3.53) and 73% patients achieved undetectable HIV RNA load (<1.7 log_10_ or 50 copies/mL). At 12-month, the median HIV RNA reduction was 2.92 log_10_ (IQR, 1.52–3.45) copies/mL and 73% patients achieved undetectable HIV RNA load. Reduction of HIV RNA levels and proportions of undetectable HIV RNA were similar irrespective of baseline HBV DNA level and HBeAg status (Table 2). Six patients had HIV RNA load above 500 copies/mL and all presented the M184I/V mutations associated with HIV resistance to 3TC. Median CD4+ T-cell counts increased from 100 (IQR: 38–178)×10^6^ cells/L at baseline to 247 (IQR: 197–374) at 12-month and 472 x10^6^ cells/L at 5 years of treatment, respectively. Median ALT was 30 (IQR: 20–39) IU/L and decreased but not significantly to 21 IU/L (IQR: 16–29) at 5 years of 3TC treatment. Kinetics of CD4+ T-cell counts and serum ALT levels in HIV-HBV co-infected patients after initiation of 3TC-containing HAART are showed in Figure 4, according to their HBeAg status. Patients with baseline CD4 T-cell counts above the median (100×10^6^ cells/L) had similar rates of undetectable levels of both HBV DNA and HIV RNA over 5 years of 3TC-containing HAART as those with CD4 T-cell counts below the median (log-rank p-values: 0.42 for HBV and 0.41 for HIV). Also, over 5-years of 3TC-containing HAART, there was no difference in increase of absolute CD4+ T-cells count among HBV virological responders and non-responders.

## Discussion

We analyzed the long-term HBV and HIV virological response in a group of 30 HIV-HBV co-infected patients, 63% HBeAg-positive, who received 3TC, as part of HAART regimen. After 12 months of 3TC-containing-HAART, the rate of HBV DNA suppression in our study was 67%, compared to the 40% reported in the international collaborative (CAESAR) study conducted in Canada, Australia, Europe and Africa [8] despite higher median HBV DNA level prior to 3TC initiation, 7.35 vs. 6.87 log_10_ IU/mL. A recent study conducted in Kenya [19] reported that 89% (17 of 19) of HIV-HBV co-infected patients achieved HBV DNA suppression (<100 IU/mL) after 18 months of 3TC treatment. The rate of HBV DNA suppression among HBeAg negative patients was 94% (17 of 18) similar to the 100% rate observed in our study. In contrast, the recent PHIDISA II study conducted among 57 HIV-HBV co-infected patients in South Africa [20] showed that HBV DNA suppression rate slight increased from 7% at baseline to 34% at 3 months of 3TC treatment and remained stable until 12 months. In the PHIDISA II study, the baseline HBV DNA level was 7.00 log_10_ IU/mL and similar to the level in our study. Furthermore, we found no relation between the baseline HIV RNA level and HBV response to 3TC-containing HAART or between the baseline HBV DNA level and HIV response to 3TC-containing HAART. Baseline CD4 count was not associated with either HBV or HIV virological response. In addition, over 5-years of 3TC-containing HAART, increases of absolute CD4+ T-cells count were not different among HBV virological responders and non-responders.

Among the 23 patients who had achieved HBV DNA suppression, 18 (78%) maintained HBV DNA suppression over a median of 51 months. This rate is much higher than the 9% reported by Benhamou et al, in HIV-HBV co-infected patients after 4 years of treatment with the same dosing of 3TC [11]. Of interest the threshold used to define HBV DNA suppression was 4.03 log_10_ IU/mL while it was 2.18 log_10_ IU/mL in our study. The higher response rate in our study may be due to a better compliance of patients to their treatment as evidenced by 73% of patients achieving HIV RNA below 50 copies/mL at 12 months and most of patients had undetectable HIV RNA, except some with high replication of drug-resistant strains. Another possibility could be that the HBV genotypes B and C are more sensitive to 3TC than genotypes A and D, which require confirmation with *in vitro* experiments. HBV DNA suppression was maintained in all HBeAg-negative patients. The higher rate of response to 3TC treatment and duration of HBV DNA suppression among HBeAg-negative patients suggest that, in resource-limited countries, HBeAg testing may be a valuable marker to predict the virological response to 3TC and could be considered when initiating in HIV-HBV co-infected patients the first-line HAART which usually includes 3TC. Other studies conducted among patients with chronic HBV infection in Japan and Brazil showed that HBeAg-negative patients had better HBV response to 3TC [21], [22].

HBeAg seroconversion and HBsAg loss are usually associated with favorable clinical outcome. Twenty-four percent (4/17) of patients lost HBsAg and 88% (7/8) of HBeAg-positive patients lost HBeAg at their last visit (median duration of 59 months). Although our study had a limited number of patients, our results are consistent with those reported in a recent study conducted in Taiwan among HBV-HIV infected patients receiving 3TC-containing HAART (median duration of 34 months), 6% (6/108) of HBsAg loss and 46% (21/46) of HBeAg seroconversion [23].

One major limitation of treating HBV with 3TC monotherapy is the rapid emergence of resistance mutations. In HBV-HIV-1 co-infected patients, resistance mutations to 3TC have been shown to occur at a rate of 15–20% per year [11], [24]. In our study, the incidence of 3TC resistance mutations detected during the first year of therapy was 3% (1of 30) which is comparable to the 7% (2 of 27) in a study conducted in Kenya (p = 0.60) [19]. Over the 5 years of follow-up, 6 of 7 patients presenting HBV breakthrough had the rtM204I (due to the ntG741A uncommon mutation) or M204V mutations associated with 3TC resistance along with rtL180M and/or rtV173L. Despite good compliance to treatment, as evidenced by the HIV RNA suppression, some patients had experienced HBV breakthrough without any mutations within the *pol* gene. This may be due to the emergence of mutations outside the *rt* domain or other mechanisms yet to identified.

A nucleotide analogue, TDF, has been shown to be active against both wild-type and 3TC resistant HBV [25], [26]. It has recently been available in Thailand at the price of 38 USD per month, which exceeded that of the current standard first line HAART (zidovudine/stavudine, 3TC and nevirapine), 30 USD per month [27], [28]. Although the Thai national [29], US [6] and WHO guidelines [30], have recommended the use of TDF+3TC or TDF+FTC as the backbone of HAART combination to treat HIV-HBV co-infected patients, this combination may not be provided to all HIV-HBV co-infected patients. Indeed, identifying HBV co-infected patients still poses a problem as shown in the study of Sungkanuparph et al where about 42% of Thai HIV-1 infected patients on ART had not been assessed for HBV co-infection [31].

In conclusion, our study shows that all HBeAg negative patients and a significant number of HBeAg positive HIV-HBV co-infected patients can achieve long-term HBV DNA and HIV suppression on 3TC containing HAART. The results of this study provide further information which may be useful for the management of HIV-HBV co-infected patients in resource-limited countries where the vast majority of HIV-HBV co-infected patients are currently receiving 3TC.

## Supporting Information

Table S1 **Summary of HBV DNA and HIV RNA loads of HIV-HBV co-infected patients on lamivudine-containing HAART.**
(DOCX)
