# Supplementary material for: Long-Term Hepatitis B Virus (HBV) Response to Lamivudine-Containing Highly Active Antiretroviral Therapy in HIV-HBV Co-Infected Patients in Thailand
Source: PLoS One. 2012 Jul 31;7(7):e42184. doi: 10.1371/journal.pone.0042184 (PMC3409123; doi:10.1371/journal.pone.0042184)
Supplement: Table S1 — Summary of HBV DNA and HIV RNA loads of HIV-HBV co-infected patients on lamivudine-containing HAART. (DOCX) [file pone.0042184.s001.docx]

**Table S1.** Summary of HBV DNA and HIV RNA loads of HIV-HBV co-infected patients on lamivudine-containing HAART

|  |  | HBV DNA load (log_10_ IU/mL) | | | | | | | HIV RNA load (log_10_ copies/mL) | | | | | | |
| --- | --- | --- | --- | --- | --- | --- | --- | --- | --- | --- | --- | --- | --- | --- | --- |
| Patient ID | Baseline HBeAg | Baseline | 3 months | 12 months | 2 years | 3 years | 4 years | 5 years | Baseline | 3 months | 12 months | 2 years | 3 years | 4 years | 5 years |
| 1 | - | 2.84 | 1.88 | 1.88 |  |  |  |  | 4.09 | 1.40 | 1.70 |  |  |  |  |
| 2 | + | 5.49 | 2.12 | 1.88 | 0.88 |  |  |  | 4.07 | 1.40 | 1.40 | 1.40 |  |  |  |
| 3 | + | 8.06 | 2.18 | 1.88 | 0.88 |  |  |  | 2.30 | 2.11 | 1.40 | 1.40 |  |  |  |
| 4 | - | 3.28 | 2.18 | 2.18 | 1.88 |  |  |  | 5.27 | 2.73 | 5.17 | 5.14 |  |  |  |
| 5 | + | 8.07 | 1.88 | 1.88 | n/a | 1.43 |  |  | 1.40 | 1.40 | 1.40 | 1.40 | 1.30 |  |  |
| 6 | + | 9.00 | 7.84 | 2.18 | n/a | 1.65 |  |  | 6.05 | 2.34 | 1.40 | 1.40 | 1.40 |  |  |
| 7 | - | 7.18 | 3.59 | 1.88 | n/a | 0.88 |  |  | 5.25 | 1.40 | 1.40 | 1.40 | 1.40 |  |  |
| 8 | - | 5.55 | 2.02 | 1.88 | n/a | n/a | 0.88 |  | 1.40 | 1.40 | 1.40 | 1.40 | 1.60 | 1.30 |  |
| 9 | + | 8.16 | 3.90 | 1.88 | n/a | n/a | 1.18 |  | 4.46 | 1.48 | 1.40 | 1.40 | 1.40 | 1.40 |  |
| 10 | - | 3.59 | 1.88 | 1.88 | n/a | n/a | 0.88 | 0.88 | 5.48 | 1.40 | 1.40 | 1.40 | 1.40 | 1.30 | 1.30 |
| 11 | - | 3.24 | 1.88 | 1.88 | n/a | n/a | 1.48 | 0.88 | 4.25 | 1.40 | 1.40 | 1.30 | 1.30 | 1.60 | 1.30 |
| 12 | - | 6.22 | 0.88 | 0.88 | n/a | n/a | n/a | 1.88 | 4.31 | 1.40 | 1.40 | 1.40 | 1.30 | 1.60 | 1.60 |
| 13 | - | 7.53 | 4.38 | 1.88 | n/a | n/a | n/a | 0.88 | 5.50 | 1.36 | 1.40 | 1.40 | 1.40 | 1.40 | 1.60 |
| 14 | - | 6.67 | 2.32 | 2.18 | n/a | n/a | n/a | 1.81 | 5.86 | 4.28 | 4.69 | 1.40 | 1.40 | 1.30 | 1.30 |
| 15 | + | 7.42 | 4.86 | 3.48 | 3.33 | 0.88 | n/a | 0.88 | 4.49 | 1.40 | 1.40 | 1.40 | 1.40 | 1.40 | 1.40 |
| 16 | + | 6.72 | 2.05 | 1.88 | n/a | n/a | n/a | 1.18 | 5.25 | 1.93 | 1.40 | 1.40 | 1.40 | 1.40 | 1.40 |
| 17 | - | 3.36 | 1.88 | 1.88 | n/a | n/a | n/a | 0.88 | 4.45 | 1.40 | 1.40 | 1.40 | 1.40 | 1.40 | 0.88 |
| 18 | - | 3.76 | 1.88 | 1.88 | n/a | n/a | n/a | 0.88 | 5.59 | 1.30 | 1.40 | 1.40 | 1.40 | 1.40 | 1.40 |
| 19 | + | 7.79 | 3.65 | 2.18 | n/a | n/a | n/a | 1.18 | 4.85 | 1.30 | 1.40 | 1.40 | 1.40 | 1.40 | 1.40 |
| 20 | + | 7.36 | 1.88 | 3.39 | 3.96 | 4.14 | n/a | 7.43 | 4.11 | 1.40 | 1.40 | 1.40 | 1.40 | 1.40 | 1.40 |
| 21 | + | 7.92 | 1.88 | 5.03 | 4.58 | 7.65 | n/a | 8.24 | 4.33 | 1.40 | 1.40 | 1.40 | 1.40 | 1.40 | 1.40 |
| 22 | + | 8.31 | 4.11 | 2.18 | 8.50 | 8.59 |  |  | 4.48 | 1.20 | 1.40 | 2.70 | 3.06 |  |  |
| 23 | + | 8.20 | 3.81 | 2.18 | 0.88 | 7.89 | n/a | 8.05 | 4.06 | 1.36 | 1.40 | 1.40 | 1.40 | 1.40 | 1.40 |
| 24 | + | 7.19 | 4.40 | 7.23 |  |  |  |  | 3.39 | 2.04 | 4.88 |  |  |  |  |
| 25 | + | 8.38 | 2.42 | 3.44 | 7.91 | 7.62 |  |  | 6.67 | 4.56 | 2.77 | 2.10 | 1.60 |  |  |
| 26 | + | 7.87 | 4.36 | 2.68 | 4.02 | 3.44 | n/a | 7.53 | 2.92 | 0.78 | 1.40 | 1.40 | 1.40 | 1.40 | 1.40 |
| 27 | + | 7.34 | 3.94 | 3.04 |  |  |  |  | 5.52 | 1.51 | 4.78 |  |  |  |  |
| 28 | + | 8.32 | 3.59 | 2.41 | 2.71 |  |  |  | 4.93 | 1.40 | 1.73 | 0.95 |  |  |  |
| 29 | + | 8.59 | 4.47 | 4.60 | 4.76 |  |  |  | 5.25 | 1.77 | 1.93 | 1.40 |  |  |  |
| 30 | + | 6.94 | 2.49 | 2.89 | 3.11 | 2.42 | 2.84 |  | 4.09 | 1.40 | 1.40 | 1.40 | 1.40 | 1.30 |  |

Note:

Patients# 1-19: controlled HBV suppression

Patients# 20-23: had experienced HBV suppression and then HBV breakthrough

Patients# 24-26: had HBV breakthrough and never reach HBV undetectable level

Patients# 27-30: never reach HBV undetectable level

Patients# 15, 26-30: had partial HBV virological response at 12 months

Patients# 20, 21, 24, 25: had experienced HBV breakthrough during the first 12 months

Patients# 1-23: were taken into account for analysis of maintaining HBV suppression
